# Hemorrhagic complications after percutaneous nephrolithotomy: angiographic diagnosis and management by transcatheter arterial embolization

**DOI:** 10.1590/0100-3984.2019.0130

**Published:** 2020

**Authors:** Vinicius Adami Vayego Fornazari, Rômulo Florêncio Tristão Santos, Thiago Franchi Nunes, Rodrigo Perrella, Tiago Magalhães Freire, Fabio Carvalho Vicentini, Joaquim Francisco de Almeida Claro, Denis Szejnfeld

**Affiliations:** 1 Radiologia Intervencionista e Cirurgia Endovascular, Escola Paulista de Medicina da Universidade Federal de São Paulo (EPM-Unifesp), São Paulo, SP, Brazil.; 2 Universidade Federal de Mato Grosso do Sul (UFMS), Campo Grande, MS, Brazil.; 3 Centro de Referência da Saúde do Homem, Hospital de Transplantes Euryclides de Jesus Zerbini, São Paulo, SP, Brazil.

**Keywords:** Nephrolithotomy, percutaneous, Aneurysm, false, Arteriovenous fistula, Embolization, therapeutic/methods, Radiology, interventional, Computed tomography angiography, Nefrolitotomia percutânea, Falso aneurisma, Fístula arteriovenosa, Embolização terapêutica, Radiologia intervencionista, Angiografia por tomografia computadorizada

## Abstract

**Objective:**

To identify the main hemorrhagic complications after percutaneous nephrolithotomy, as well as the results obtained with transcatheter arterial embolization (TAE) at an interventional radiology center.

**Materials and Methods:**

This was a retrospective analysis of patients undergoing TAE for the treatment of hemorrhagic complications after percutaneous nephrolithotomy. All patients underwent computed tomography angiography (CTA).

**Results:**

We evaluated a total of nine patients. At emergency department readmission, the most common symptom was macroscopic hematuria, which was seen in five patients. Three patients had an isolated pseudoaneurysm, two had a pseudoaneurysm together with active bleeding (perirenal hematoma), and one had a pseudoaneurysm together with arteriocalyceal fistula. Arteriovenous fistula was diagnosed in three patients and was not seen in combination with other vascular lesions. We did not identify arteriocalyceal fistula in isolation. Five patients underwent TAE with 6 × 15 mm and 6 × 20 mm microcoils. Four patients underwent TAE with n-butyl-2-cyanoacrylate and ethiodized oil. Follow-up CTAs revealed no complications.

**Conclusion:**

Because of its high diagnostic accuracy, CTA provides the interventional radiologist with valuable data for individualized therapeutic planning. The TAE procedure is safe and effective. It can therefore be used as a first-line treatment for hemorrhagic complications resulting from percutaneous renal procedures.

## Abstract (pt)

**Objetivo:**

Demonstrar as principais complicações hemorrágicas após nefrolitotripsia percutânea, bem como os resultados após o tratamento por embolização arterial transcateter (EAT) em um centro de radiologia intervencionista.

**Materiais e Métodos:**

Coleta e análise de dados retrospectivos de pacientes submetidos a EAT por complicações hemorrágicas após nefrolitotripsia percutânea.

**Resultados:**

O sintoma mais comum foi hematúria macroscópica, presente em cinco pacientes no momento da readmissão ao pronto-socorro, e nestes pacientes identificamos três pseudoaneurismas isolados, dois casos de combinação de pseudoaneurisma e sangramento ativo (hematoma perirrenal) e um caso de associação de pseudoaneurisma e fístula arteriocalicinal. Fístula arteriovenosa foi diagnosticada em três pacientes, não sendo observada em associação com outras lesões vasculares. Não identificamos fístula arteriocalicinal isolada, somente associada a pseudoaneurisma. Cinco pacientes foram submetidos a embolização por micromolas 6 × 15 mm e 6 × 20 mm. Quatro pacientes foram submetidos a embolização por Histoacryl e Lipiodol. Não observamos complicações pela angiotomografia computadorizada de controle.

**Conclusão:**

A angiotomografia computadorizada apresenta alta acurácia diagnóstica e guarnece o radiologista intervencionista de dados para um planejamento terapêutico individualizado. EAT é um procedimento seguro e eficaz e pode ser utilizado como primeira linha para o tratamento de complicações hemorrágicas resultantes de procedimentos percutâneos renais.

## INTRODUCTION

Percutaneous nephrolithotomy (PCNL) is the procedure of choice for the treatment of kidney stones larger than 2 cm^(1)^. Although percutaneous renal surgery is less invasive than conventional surgery, there can be complications, the most worrisome of which is renal hemorrhage, which requires a blood transfusion in 1-11% of cases^(2,3)^.

Iatrogenic renal vascular lesions that form pseudoaneurysms, arteriovenous fistulas (AVFs), or arteriocalyceal fistulas (ACFs) are rare but life-threatening conditions. Generally, the main symptomology includes macroscopic hematuria, although there are a variety of other signs or symptoms, such as flank pain, nausea, vomiting, dizziness, and fever. The severity of bleeding varies; some patients can progress rapidly to hemodynamic instability and heart failure^(4,5)^. It is thought that these lesions stem from the transection of an artery during the percutaneous procedures^(5)^.

Catheter-directed digital subtraction angiography (CDSA) can be considered the gold standard for the evaluation of patients with iatrogenic renal vascular lesions, because it presents diagnostic efficacy in the identification of bleeding^(4)^. In most cases, conservative treatment is successful in stabilizing such patients. However, when the bleeding is persistent or hemodynamically significant, intervention in the form of transcatheter arterial embolization (TAE) is needed, being employed in 0.8-1.4% of cases^(2,4)^. In the past, angiography required high doses of iodinated contrast media with high osmolarity, which translated to an increased risk of contrast-induced nephropathy. After the introduction of low-osmolarity and iso-osmolarity contrast media, as well as the improvements made to the devices and the development of highly selective TAE techniques, that risk became insignificant^(5)^. With the concomitant evolution of diagnostic imaging methods and the creation of the interventional radiologist specialty, acute bleeding can now be diagnosed with precision and treated effectively^(6)^, making digital subtraction angiography followed by TAE the method of choice for the diagnosis and treatment of acute hemorrhagic complications^(1,2,4-6)^. Therefore, knowledge of iatrogenic vascular complications after PCNL can help interventional radiologists plan and execute the TAE.

In the present study, we describe a series of iatrogenic renal vascular complications after PCNL. We also present the results obtained with selective TAE.

## MATERIALS AND METHODS

This study was approved by the local committee for ethics in research and in the management of teaching and research. Because of the retrospective nature of the study, the requirement for informed consent was waived.

### Selection of patients

We evaluated data referring to iatrogenic renal vascular lesions after PCNL, treated with selective TAE by the interventional radiology team at our institution between February 1, 2013 and June 30, 2019. The clinical and surgical data were extracted from the prospective database of the department of endourology and renal calculi. Patients who underwent TAE for other causes were excluded. Patients were assigned numbers to ensure the confidentiality of information and to protect their privacy.

### Collection and data analysis

**Figure 1:**
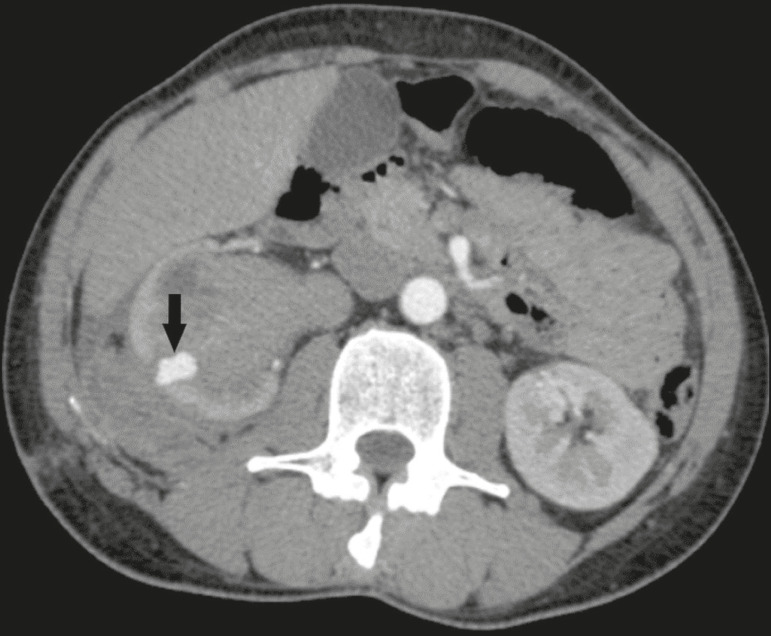
Axial CTA, in the corticomedullary phase, showing a pseudoaneurysm in the renal middle segment and a perirenal hematoma on the right.

The data collected were analyzed retrospectively. We evaluated information on age and gender, as well as on signs and symptoms at emergency department readmission, together with information such as the presence or absence of congestive heart failure and pulmonary embolism. We analyzed the initial computed tomography angiography (CTA) images (Figure 1) performed on hemodynamically stable patients, acquired in order to detect iatrogenic vascular complications, as well as a to confirm and discriminate the type of vascular lesion (pseudoaneurysm, high-flow AVF, low-flow AVF, or ACF), and attempted to determine whether those lesions were solitary or were accompanied by other lesions. Other variables were also analyzed: the number and size of pseudoaneurysms; the number of AVFs; the number of ACFs; the location of the lesion; perirenal hematoma; free fluid in the abdomen; the type of embolic material used; and the length of hospital stay.

Parenchymal loss was evaluated by comparing the CDSA images obtained before and after TAE. The CDSA images were evaluated by three interventional radiologists, working independently, and disagreements were resolved by consensus. The amount of parenchyma lost after TAE was categorized into four groups, by proportion: < 5%, 5-10%, 10-25%, and 25-50%. For each CDSA, that proportion was determined by calculating the relationship between the area with no contrast uptake and the total area of the kidney. Initial and late complications were evaluated, and a follow-up CTA was performed.

### Embolization procedure

**Figure 2:**
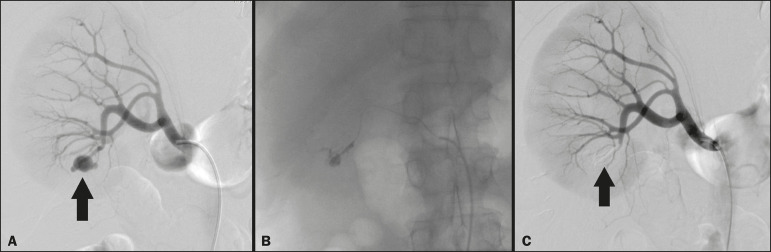
**A:** Pre-embolization CDSA showing a pseudoaneurysm in the lower third of the right kidney. **B:** Selective catheterization and embolization of the affected segment. **C:** Post-embolization arteriography showing the absence of a pseudoaneurysm and no signs of immediate complications.

**Figure 3:**
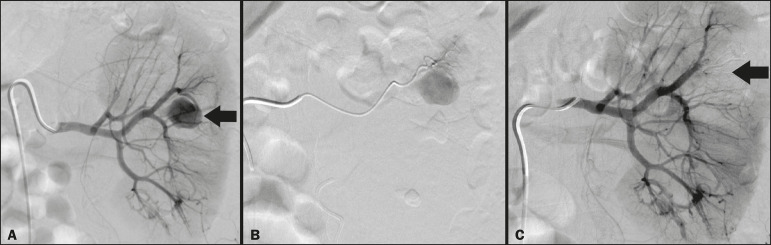
**A:** Pre-embolization CDSA showing a pseudoaneurysm in the middle segment of the left kidney. **B:** Selective catheterization and embolization of the affected segment. **C:** Post-embolization arteriography showing the absence of a pseudoaneurysm and no signs of immediate complications.

**Figure 4:**
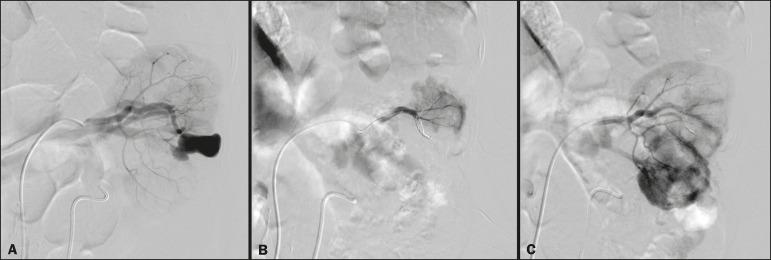
**A:** Pre-embolization CDSA showing an ACF in the middle segment of the left kidney. **B:** Selective catheterization and embolization of the affected segment. **C:** Post-embolization arteriography showing the absence of an ACF and no signs of immediate complications.

The selective TAE procedures were executed by two interventional radiologists with 10 and 15 years of experience, respectively. Arteriography was performed with femoral artery access under local anesthesia. After obtaining vascular access with a 5 Fr sheath (Cobra; Cordis/Cardinal Health, Dublin, OH, USA), we performed selective catheterizations, together with CDSA of the renal arteries. The interlobar arteries causing the bleeding were detected, and selective catheterization was performed with 2.7 Fr microcatheters (Progeat; Terumo Corporation, Tokyo, Japan). The pseudoaneurysms, AVFs, and ACFs were selectively embolized using microcoils or n-butyl-2-cyanoacrylate (Histoacryl; B. Braun, Melsungen, Germany) with ethiodized oil (Lipiodol; Guerbet, Villepinte, France), in order to occlude the arterial flow to the lesion with minimal renal parenchymal loss (Figures 2, 3, and 4). The embolization was concluded when the blood flow to the lesion could no longer be identified on the CDSA. The iodinated contrast media concentration used was 300 mg/mL, and the maximum volume of contrast media administered was 2 mL/kg of body weight.

There are no clear contraindications to embolization of urgent bleedings, because the procedure is therapeutic, even in cases of blood dyscrasias, in which the bleeding at the access site can be stanched with vascular sealants.

### Technical and clinical success

The technical success of the procedure has been defined as complete embolization of the pseudoaneurysms, AVFs, and ACFs, as verified in a follow-up CTA images obtained between four and six weeks after the TAE. Clinical success has been defined as hemodynamic stability, improvement of hematuria, and preservation of renal function, over the first 60 days after the procedure (during hospitalization and outpatient care).

### Statistical analysis

The data were entered into an Excel spreadsheet and exported to the SPSS statistics software, version 20.0 (IBM Corp., Armonk, NY, USA) for statistical analysis.

## RESULTS

Of 1133 patients who had undergone PCNL, nine (0.79%), all of whom who had undergone TAE, presented with renal vascular lesions after PCNL and were therefore included in the study. There were five males and four females, and the mean age of the patients in the sample was 53 years (range, 34-75 years). Data related to the operative time, site of puncture, hemoglobin (pre- and post-PCNL), the need for blood transfusion, and the post-PCNL change in the serum creatinine level are shown in Table 1. Table 2 shows the demographic characteristics (age and gender), as well as the clinical characteristics (signs and symptoms at emergency department readmission; and the presence or absence of congestive heart failure and of pulmonary embolism) of the sample.

**Table 1:** Data related to the surgical procedure (PCNL).

Patient	Operative time	Laterality	Location of puncture (calyx)	Supracostal puncture	Number of punctures	Hemoglobin pre-PCNL (g/dL)	Hemoglobin post-PCNL (g/dL)	Transfusion	ΔSerum creatinine (mg/dL)
1	101 min	Right	Lower	Yes	2	12.1	10.5	No	0.2
			Upper						
2	80 min	Right	Lower	No	1	11.2	8.1	Yes, before endovascular treatment	1.0
3	90 min	Right	Lower	No	2	11.0	8.8	Yes, before endovascular treatment	0.8
			Middle						
4	130 min	Left	Lower	Yes	2	13.0	11.1	No	0.3
			Upper						
5	50 min	Right	Lower	No	1	13.9	11.8	No	0.5
6	60 min	Left	Middle	No	1	13.6	11.9	No	0.5
7	60 min	Left	Lower	Yes	2	14.1	12.4	Yes, before endovascular treatment	0.7
			Middle						
8	185 min	Right	Lower	Yes	4	13.8	9.7	Yes, before endovascular treatment	0.8
			Middle						
			Middle						
			Upper						
9	135 min	Right	Lower	Yes	3	12.0	10.2	No	0.8
			Lower						
			Upper						

**Table 2:** Demographic and clinical characteristics of patients with post-PCNL iatrogenic renal vascular lesions treated with selective TAE

Patient	Age (years)	Gender	Symptoms and indication for CTA	CHF or pulmonary embolism
1	42	Male	Macroscopic hematuria	Absent
2	75	Male	Acute flank pain	Absent
3	55	Female	Macroscopic hematuria	Absent
4	64	Male	Macroscopic hematuria	CHF
5	34	Male	Drop in hemoglobin	Absent
6	61	Female	Acute flank pain	Absent
7	53	Female	Macroscopic hematuria	Absent
8	44	Male	Macroscopic hematuria	Absent
9	49	Female	Acute flank pain	Absent

CHF, Congestive heart failure.

The most common symptom was macroscopic hematuria, which was present in five patients at emergency department readmission. In the immediate postoperative period, three patients presented acute flank pain and one patient presented a drop in hemoglobin levels. Among the patients in the study sample, we identified three isolated pseudoaneurysms, two cases of pseudoaneurysm with active bleeding (perirenal hematoma), and one case of pseudoaneurysm with ACF. No pseudoaneurysm together with AVF was observed. An isolated AVF was diagnosed in three patients. We identified no cases of ACF without pseudoaneurysm. None of the patients presented all three types of vascular lesions together. The iatrogenic lesions were in the right kidney in six of the patients and in the left kidney in the three remaining patients. Two of the lesions were in the upper pole, four were in the middle segment, and three were in the lower pole.

In the present study, the number of pseudoaneurysms was one per patient, and the mean pseudoaneurysm size was 19.6 mm (range, 13-30 mm). The number of AVFs was also one per patient, as was the number of ACFs. Five patients underwent embolization with 6 × 15 mm and 6 × 20 mm microcoils, two or three coils being used in each patient. Four patients underwent embolization with Histoacryl and Lipiodol, in a 1:3 mixture, until the pseudoaneurysm was filled or the arterial branch was occluded, the quantity of embolic material never exceeding 10 mL. The proportion of parenchyma lost was < 5% in four patients, 5-10% in four patients, and 10-25% in one patient. No parenchymal losses greater than 25% were observed. Four patients needed blood transfusions before the endovascular procedure, and none of the patients received a blood transfusion after TAE. The mean volume of iodinated contrast media used for the TAE was 70 mL (range, 40-80 mL). The length of hospital stay after selective TAE was 2-11 days. No immediate or late complications were identified on the CTA. None of the patients required a second embolization procedure for rebleeding during the 60-day follow-up period. Table 3 shows the type of vascular lesion, the location of the lesion within the kidney, the number/size of the lesions, the embolic material used, and the proportion of renal parenchyma lost after embolization.

**Table 3:** Characteristics of the iatrogenic renal vascular lesions and of the embolization procedures used in their treatment.

Patient	Iatrogenic renal vascular lesion(s)				Embolization procedure	
	Type	Location	Number and size		Agents employed	Parenchymal loss
1	Low flow AVF	Right kidney, lower pole	1 AVF		Histoacryl + Lipiodol	10–25%
2	Pseudoaneurysm and perirenal hematoma	Right kidney, lower pole	1 pseudoaneurysm (22 mm)		Histoacryl + Lipiodol	5–10%
3	Pseudoaneurysm and ACF	Right kidney, middle segment	1 pseudoaneurysm (13 mm), 1 AVF		3 coils (6 × 20 mm)	< 5%
4	High flow AVF (CHF)	Left kidney, middle segment	1 AVF		2 coils (6 × 20 mm)	5–10%
					2 coils (6 × 15 mm)	
5	Low flow AVF	Right kidney, upper pole	1 AVF		2 coils (6 × 15 mm)	< 5%
6	Pseudoaneurysm	Left kidney, upper pole	1 pseudoaneurysm (30 mm)		Histoacryl + Lipiodol	5–10%
7	Pseudoaneurysm and perirenal hematoma	Left kidney, middle segment	1 pseudoaneurysm (15 mm)		2 coils (6 × 15 mm)	< 5%
8	Pseudoaneurysm	Right kidney, middle segment	1 pseudoaneurysm (18 mm)		3 coils (6 × 15 mm)	< 5%
9	Pseudoaneurysm	Right kidney, lower pole	1 pseudoaneurysm (20 mm)		Histoacryl + Lipiodol	5–10%

CHF, congestive heart failure.

## DISCUSSION

Since the first PCNL, performed in 1976, the procedure has become the standard intervention and has been replacing conventional surgery for the treatment of renal lithiasis^(1,4)^. Although PCNL is minimally invasive, complications can occur during the passage of the needle, during dilatation of the urinary tract, during the nephroscopy, or even in the postoperative period^(2,4)^. Hemorrhage is a common complication, with a reported incidence of up to 6%^(1)^. Bleeding occurs most commonly due to lesion of the anterior or posterior segmental arteries, which can be prevented by performing a posterolateral renal puncture, along the avascular plane known as Brödel’s line^(7,8)^. Clinically, it can manifest as macroscopic hematuria, with variable severity of bleeding, some patients progressing rapidly to hemodynamic instability and (in cases of a high-flow AVF) heart failure^(2,4,5)^. Although the time from the renal intervention to the appearance of iatrogenic renal vascular lesions can vary, complications generally occur within the first three weeks after the PCNL.

In the case of renal arterial lesion, hemostatic mechanisms such as reduction of blood flow, formation of blood clots, and pressure from adjacent tissues are activated in order to control the bleeding. When these mechanisms fail and the blood flow to the damaged artery increases, blood leakage can form a pseudoaneurysm, which is a blood collection confined to the side of the vessel by surrounding tissue. In the present study, the most common iatrogenic renal vascular lesion was pseudoaneurysm, which was observed in six of the nine patients. A true aneurysm is distinguished from a pseudoaneurysm by its involvement of all the three layers of the arterial wall (intima, media, and adventitia). When a pseudoaneurysm becomes larger and blood leaks into the renal collecting system, macroscopic hematuria can occur. In the literature, macroscopic hematuria, with or without flank pain, has been reported in nearly all cases of pseudoaneurysm^(4,9)^.

An AVF is an abnormal communication between arterial and venous systems that are in close proximity, causing a fistulous connection, without an intermediary capillary bed^(4,5)^. An AVF may be asymptomatic but can also cause macroscopic hematuria, hypertension, or (in cases of a high-flow AVF) heart failure^(4)^.

The true incidence of the combination of pseudoaneurysm and AVF remains unknown. Among the patients evaluated in the present study, that combination was not observed. A fistula to the collecting system can also occur, and an ACF is an abnormal communication between the arterial system and the renal pelvis, with potentially serious clinical bleeding. In the present study, none of the patients presented an ACF in isolation and one patient presented ACF together with a pseudoaneurysm. Postoperative bleeding certainly can occur separately from these processes, usually leading to the formation of a perirenal hematoma, which is typically venous and does not involve a fistula to the collecting system, typically resolving spontaneously, although it can cause a postoperative reduction in hematocrit.

For all the patients in our sample, the predominant symptom at presentation of the iatrogenic renal vascular lesion was macroscopic hematuria. That is in keeping with the findings of other studies of the topic in the literature, in which most patients also presented with macroscopic hematuria, although there have also been many reports of patients presenting with flank pain, nausea, vomiting, dizziness, fever, or simply a reduction in hematocrit^(4,5)^. Four patients in our sample required a blood transfusion, a fact that is probably due to the timely, effective intervention with TAE.

Angiography is one of the first radiologic examinations suggested for the diagnosis of iatrogenic renal vascular lesions. In emergencies, CTA has advantages over other imaging modalities. The arterial and nephrographic phases of the CTA should be preferably performed to detect renal iatrogenic vascular lesions^(1)^. In the acute-abdomen protocol employed at our institution, CTA is the initial diagnostic method.

All health professionals should be aware of the potential post-PCNL complications. In a postoperative PCNL patient with signs and symptoms characteristic of such complications, especially macroscopic hematuria, CTA facilitates the diagnosis of hemorrhagic complications and the planning of the procedure, allowing a targeted study that reduces not only the procedure time but also the doses of contrast media and radiation, as well as facilitating the choice of endovascular device that is most well suited for each case. At facilities with angiography equipment capable of tomographic application and three-dimensional reconstruction, as well as a wide array of endovascular materials, an initial CTA evaluation can be excluded.

Before TAE, clinical management and conventional surgery were the only treatment options for acute bleeding. Currently, TAE is one of the first treatment options for such bleeding^(2,4,5,9)^. A successful renal artery embolization is defined as the total and permanent closure of the damaged renal arterial branch. In a study of 4,695 patients undergoing PCNL, Richstone et al.^(2)^ reported that 57 (1.2%) required selective TAE postoperatively. In a study of 1,854 patients, Srivastava et al.^(3)^ reported that, after PCNL, TAE was required in 24 patients, an isolated pseudoaneurysm was observed in 13, and pseudoaneurysm combined with AVF was observed in four.

The areas of the parenchyma in which iatrogenic renal vascular lesions occur already theoretically present a significant percentage of pre-embolization renal infarction, resulting in impaired renal function. The selectivity of the embolization allows the renal infarction to be minimized, thereby maximizing the preservation of the parenchyma. In our sample, the renal function was well preserved after embolization. Shapiro et al.^(9)^ reported “stable” renal function in six patients who had undergone TAE for the treatment of a pseudoaneurysm. In a study conducted by Martin et al.^(10)^, no significant difference was found between pre-embolization and post-embolization serum creatinine levels^(10)^.

Difficulties in embolization procedures include renal failure, tortuous blood vessels, and renal artery stenosis^(4,11)^. In the case of renal artery stenosis, embolization can be performed after angioplasty of the renal artery. Complications of TAE, such as renal artery dissection, post-embolization syndrome, and loss of renal function, which are extremely rare^(4,12-15)^, did not occur in our study sample.

A TAE is performed selectively or highly selectively, and the choice of the embolic agent (autologous blood clot, detachable balloons, coils, particles, or Histoacryl) is made according to the interpretation of the interventional radiologist, who must take into consideration the caliber/branching the vessel, the blood flow (high or low), the drainage, and the venous or lymphatic contribution. In the present study, the agents of choice were a coil with controlled deployment and Histoacryl. Typically, only one session of TAE is needed^(4)^.

In our study, the five patients with life-threatening hematuria underwent CDSA and TAE. None of the patients presented with recurrence that would have necessitated a second TAE. In the case of persistent bleeding or rebleeding, another CDSA and TAE can be performed, depending on the clinical condition of the patient and the laboratory test results. The rate of success of TAE on a first attempt was 100%, similar to that reported in other studies^(6)^, although the small size of our patient sample should be taken into consideration.

Our study has some limitations. First, it was retrospective and included a relatively small number of cases. However, all the cases were extracted from a prospective database of a high-volume facility, with evaluation by CTA before and after the procedure, In addition, the clinical and radiological evaluations were well documented.

The objective of this study was to evaluate iatrogenic renal vascular lesions after PCNL, their management using selective TAE, and the results of this procedure. The initial results were positive, showing a high rate of success. In the follow-up period after TAE, there was no loss of renal function and no rebleeding in any of the patients.

## CONCLUSION

Renal hemorrhagic complications should be considered in patients with macroscopic hematuria after percutaneous renal interventions, and the most common findings in our sample were pseudoaneurysm and AVF. In addition to having high diagnostic accuracy, CTA provides the interventional radiologist with data for an individualized therapeutic planning. The TAE procedure is safe and effective and can be used as a first-line treatment for hemorrhagic complications resulting from percutaneous renal procedures.
